# Histological image segmentation using fast mean shift clustering method

**DOI:** 10.1186/s12938-015-0020-x

**Published:** 2015-03-20

**Authors:** Geming Wu, Xinyan Zhao, Shuqian Luo, Hongli Shi

**Affiliations:** School of Biomedical Engineering, Capital Medical University, Beijing, China; Liver Research Center, Beijing Friendship Hospital, Capital Medical University, Beijing, China

**Keywords:** Clustering, Colour image segmentation, Mean shift, Histological image processing

## Abstract

**Background:**

Colour image segmentation is fundamental and critical for quantitative histological image analysis. The complexity of the microstructure and the approach to make histological images results in variable staining and illumination variations. And ultra-high resolution of histological images makes it is hard for image segmentation methods to achieve high-quality segmentation results and low computation cost at the same time.

**Methods:**

Mean Shift clustering approach is employed for histological image segmentation. Colour histological image is transformed from RGB to CIE L*a*b* colour space, and then a* and b* components are extracted as features. To speed up Mean Shift algorithm, the probability density distribution is estimated in feature space in advance and then the Mean Shift scheme is used to separate the feature space into different regions by finding the density peaks quickly. And an integral scheme is employed to reduce the computation cost of mean shift vector significantly. Finally image pixels are classified into clusters according to which region their features fall into in feature space.

**Results:**

Numerical experiments are carried on liver fibrosis histological images. Experimental results demonstrate that Mean Shift clustering achieves more accurate results than k-means but is computational expensive, and the speed of the improved Mean Shift method is comparable to that of k-means while the accuracy of segmentation results is the same as that achieved using standard Mean Shift method.

**Conclusions:**

An effective and reliable histological image segmentation approach is proposed in this paper. It employs improved Mean Shift clustering, which is speed up by using probability density distribution estimation and the integral scheme.

## Background

In recent years, with the increasing demands of quantitative analysis, digital image processing techniques attract more and more attention in histopathology [1,2]. They are considered to be more reliable than traditional manual assessment which heavily depends on the operator’s experience and usually can not be reproduced. Image segmentation serves as a fundamental and key technique and is typically the first step in digital image analysis. In histological image analysis, it partitions a digitized histological image into multiple homogeneous or similar regions which corresponding to different tissues or cellular components. As the result of image segmentation is taken as the input of the successive processing steps, it is essential and critical to the quality of final result of qualitative image analysis.

Histological image segmentation is much difficult due to the complex approach to make histological images. Histological specimens are fixed, processed, embedded, sectioned and then stained into different colours in different tissues. Coloured histological sections provide anatomical details for the diagnosis and histopathology, and bring out special features of different tissues at the microscopic level. To identify different tissues or cellular components, histological sections are segmented according to colour, shape or texture features after acquired with high-resolution digital camera, and then classified by commonly employing supervised methods [3-6]. Several methods based on digital image processing and pattern recognition techniques have been proposed to deal with histological image segmentation problem in the past years [7-12]. When no training data set is available in histological image analysis, feature vectors or points representing pixels in the histological image are usually extracted from their local properties. Then unsupervised techniques are used to label pixels into different clusters by separating feature vectors in the feature space. There are many existing approaches in literature which can be employed for segmenting histological image. These include clustering based methods (k-Means [13] and Mean Shift [14]), mixture models based schemes (i.e. Gaussian mixture models based Expectation-Maximization clustering, GMM-EM [15]) and state-of-the-art energy minimization based approaches (graph-cuts methods [16-18] and Markov Random Field based method [19]). However, histological image segmentation still faces several technique challenges. One of them is how to achieve high-quality segmentation results with low computation cost, especially for sequential histological image analysis. The size of acquired histological image is usually very large for better investigation of micro-structures. It makes most of existing image segmentation algorithms, such as Mean Shift, very time-consuming and hard to be used in practice. Mean Shift is a non-parametric clustering approach which has no assumptions on the shape of the distribution and the number of clusters. So Mean Shift may achieve better segmentation results than model-based clustering schemes when it is used as a histological image segmentation method.

In this paper, we focus on pixel-level segmentation by colours in histological image with unsupervised method. A fast Mean Shift clustering approach is proposed and applied to segment histological images properly. The new method estimates the probability density distribution in advance and then separates the feature space into different regions by employing Mean Shift to find the density peaks. Feature points are divided into clusters according to the region that they fall into. And an integral scheme is employed to speed up the computation of mean shift vector. To apply the proposed method to histological image segmentation, CIE L*a*b* colour space is used as feature space.

The rest parts of this article are organized as follows: Section 2 describes the main ideas and schemes employed to speed up Mean Shift clustering approach in details. The framework of histological image segmentation based on the proposed method is also given in this section. Section 3 shows numerical experimental results from live fibrosis histological images, and followed by the study conclusion in Section 4.

## Methods

### Feature extraction in L*a*b* color space

To obviously identify different tissues in histological specimens, they are stained into different colours. Digitized histological images are usually acquired and stored in RGB. CIE L*a*b* colour space is a non-linear transformation of RGB, and models all visible colours approximate to human vision. So the simple Euclidean distance in L*a*b* space could differentiate among colours perceptually. We convert histological images from RGB space to L*a*b* space and only extract a* and b* components as features for clustering to ignore variations in brightness. Thus histological image segmentation becomes a 2D clustering problem. Denote *V*_*ab*_ the feature space consisting of a* and b* components.

### Fast mean shift clustering

Given a set of points {*x*_1_,*x*_2_,…*x*_*n*_} in *V*_*ab*_, mean shift vector (MSV) with uniform kernel is defined as follows

1 $$ \begin{array}{cc}\hfill {m}_h(x)=\frac{1}{K_S}{\displaystyle \sum_{x_i\in {S}_h(x)}\left({x}_i-x\right)},\hfill & \hfill \forall x\in {V}_{ab}\hfill \end{array}, $$

where *S*_*h*_ is a sphere with center *x* and radius *h*, and *K*_*s*_ is the number of points located in *S*_*h*_. *h* is termed the window size.

Standard Mean Shift clustering method employs an iterative gradient ascent procedure to estimate local density. For a given point *x* in the feature space, it sets *x* ← *x* + *m*_*h*_(*x*) and repeats this step until convergence. The stationary points of this procedure represent the modes of the underlying distribution. Points associated with the same stationary point are considered as members of the same cluster.

#### Mean shift vector estimation

To find out the mean shift vector at *x*, one has to compute the distances from all points to the specified centre *x* and selects out those located in *S*_*h*_. Thus the computational cost of mean shift vector is very high and results in standard Mean Shift a very slow clustering approach.

**Figure 1:**
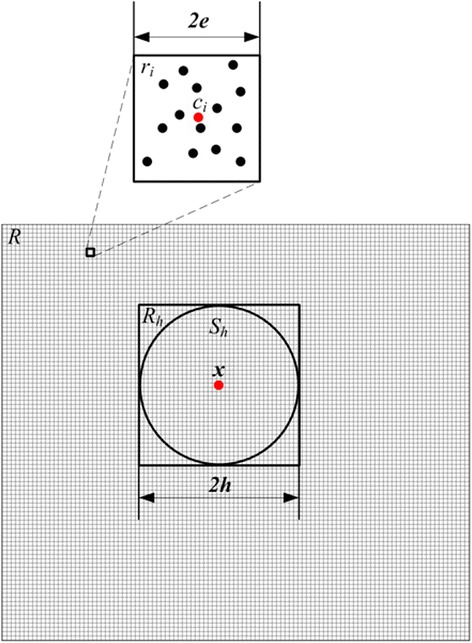
**The relationship of** ***S***_***h***_ **and** ***R***_***h***_ **in two dimension case.** Black points represent observations. The region *R* is split into tiny squares which are used to represent the observations located in them.

Notice that all points are located in a square *R* in *V*_*ab*_. Split *R* into tiny squares {*r*_1_,*r*_2_*,…,r*_*m*_ whose sides have the length of 2*e*. For each tiny square *r*_*i*_, denote the frequency of points in it by *w*_*i*_ and the center by *c*_*i*_. Denote the closest external square of *S*_*h*_ by *R*_*h*_ which consists of tiny squares (Figure 1 illustrates the relationship of *S*_*h*_ and *R*_*h*_ in *V*_*ab*_). We replace *R*_*h*_ with *R*_*h*_ and approximate *m*_*h*_ at *x* with

2 $$ {\widehat{m}}_h(x)=\frac{1}{K_R}{\displaystyle \sum_{x_i\in {R}_h(x)}\left({x}_i-x\right)}. $$

Here, *K*_*R*_ is the number of observations located in *R*_*h*_, and now *h* is half of the side length of *R*_*h*_ in what follows. Considering the definition of *w*_*i*_, we can rewrite (2) as

3 $$ {\widehat{m}}_h(x)=\frac{{\displaystyle \sum_{x_i\in {R}_h(x)}{x}_i}}{{\displaystyle \sum_{r_i\in {R}_h(x)}{w}_i}}-x. $$

Given *e* ≪ *h*, we have a further approximation

4 $$ {\widehat{m}}_h(x)\approx \frac{{\displaystyle \sum_{r_i\in {R}_h(x)}{w}_i{c}_i}}{{\displaystyle \sum_{r_i\in {R}_h(x)}{w}_i}}-x. $$

If *w*_*i*_ and *c*_*i*_ are computed in advance and stored in the frequency matrix *w* and the centre matrix *c* respectively, *R*_*h*_ corresponds to a sub-region of these matrices. Thus for a given *x*, it is not needed to find out the points located in *S*_*h*_ by computing the distances from all points to the *x*. So the computation cost of ${\widehat{m}}_h$ is much lower than that of *m*_*h*_.

#### Using of integral image scheme

We employ the above method to estimate mean shift vector in a*b* colour space. For the convenience of description, we use two subscripts to index variants defined in the above section. Rewrite Eq. (4) as follows

5 $$ {\widehat{m}}_h(x)\approx \frac{{\displaystyle \sum_{\begin{array}{l}m-h\le i\le m+h\\ {}n-h\le j\le n+h\end{array}}w\left(i,j\right)c\left(i,j\right)}}{{\displaystyle \sum_{\begin{array}{l}m-h\le i\le m+h\\ {}n-h\le j\le n+h\end{array}}w\left(i,j\right)}}-x, $$

where (*m*,*n*) satisfies ‖*c*(*m*, *n*) − *x*‖_∞_ ≤ *e*.

**Figure 2:**
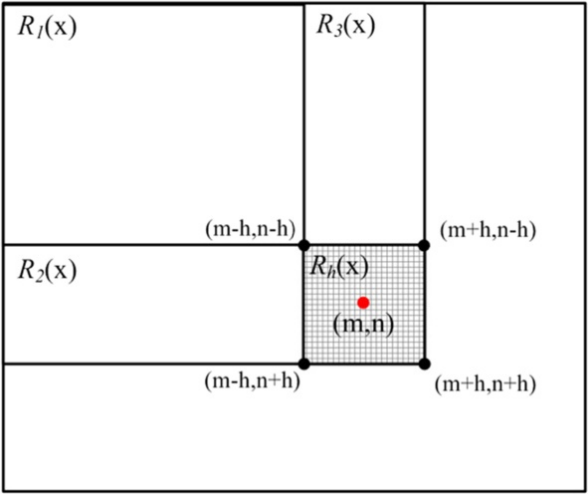
**Integral image scheme used to speed up the computation of mean shift vector.** The sum of elements within the region *R*_*h*_ can be simply computed by using four integral image values at its four corners.

Notice that the sum operations in Eq. (5) are over all elements in a specified squared sub-region. So the integral image scheme can be employed to speed up the computation of ${\widehat{m}}_h$. Integral image, also known as summed area table, was proposed for texture mapping in [20] and then widely used in pattern recognition and image processing [21,22]. It can be used to rapidly calculate summations over sub-regions of an image. The value at location (*i*,*j*) in the integral image is defined as the sum of all elements within the top and left side of (*i*,*j*). As Figure 2 shows, the sum of elements within the region *R*_*h*_ can be simply computed by using four integral image values at its four corners.

To employ the integral image scheme, we define

6 $$ W\left(k,l\right)={\displaystyle \sum_{i\le k,j\le l}w\left(i,j\right)} $$

and

7 $$ M\left(k,l\right)={\displaystyle \sum_{i\le k,j\le l}w\left(i,j\right)c\left(i,j\right)}. $$

Then we obtain

8 $$ {\displaystyle \sum_{\begin{array}{l}m-h\le i\le m+h\\ {}n-h\le j\le n+h\end{array}}w\left(i,j\right)c\left(i,j\right)}=M\left(m+h,n+h\right)+M\left(m-h,n-h\right)-M\left(m+h,n-h\right)-M\left(m-h,n+h\right) $$

and

9 $$ {\displaystyle \sum_{\begin{array}{l}m-h\le i\le m+h\\ {}n-h\le j\le n+h\end{array}}w\left(i,j\right)}=W\left(m+h,n+h\right)+W\left(m-h,n-h\right)-W\left(m+h,n-h\right)-W\left(m-h,n+h\right). $$

Thus mean shift vector can be approximately computed as following

10 $$ {\widehat{m}}_h(x)\approx \frac{M\left(m+h,n+h\right)+M\left(m-h,n-h\right)-M\left(m+h,n-h\right)-M\left(m-h,n+h\right)}{W\left(m+h,n+h\right)+W\left(m-h,n-h\right)-W\left(m+h,n-h\right)-W\left(m-h,n+h\right)}-x. $$

The above formula shows that the computation cost of mean shift vector is no longer related to the number of points and can be performed in constant time.

### Histological image segmentation framework

Based on the above discussion, the framework of the proposed fast mean shift method (FMShift) for histological image segmentation is summarized as follows:

Algorithm: Fast mean shift clustering for histological image segmentation

Initialization:

Input color histological image *I*, *e* and *h* (≫*e*).

Main:

Step 1: Transform *I* from RGB to L*a*b* color space, and extract a* and b* color components to build a feature space *V*_*ab*_. Then separate the feature space into tiny squares with side length of 2*e*, and compute the frequency matrix *w* of feature points and center matrix *c*.

Step 2: Compute *W* and *M* by using Eq. (6) and (7). Employ Mean Shift approach to separate the feature space into different regions by finding the density peaks.

Step 3: Divide pixels of histological image into clusters according to which region their corresponding feature point falls into.

Output:

Segmentation labels

## Experiments and discussion

**Figure 3:**
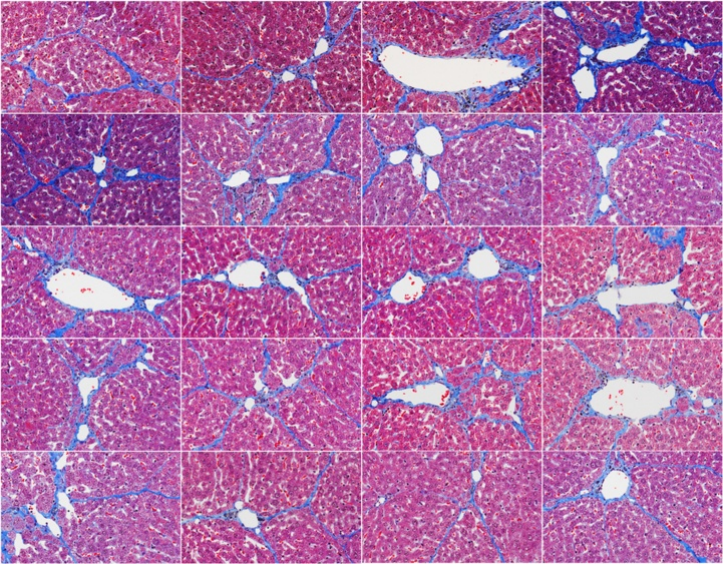
**Original liver fibrosis histological images for performance evaluation.** Twenty histological images obtained for performance evaluation by digitizing several sections from a liver histological specimen of a Wistar rat.

Quantitation of connective tissue and collagen is important in the assessment of fibrosis progression in chronic liver diseases. To evaluate our proposed method, a liver histological specimen from a Wistar rat, in which liver fibrosis was induced by albumin antigen-antibody complex, was used for the evaluation. With the use of Masson’s trichrome staining, connective tissue and collagen were stained in light blue while smooth muscle in red and nucleus of hepatocytes in light dark. And blood vessels and sinusoids are in white. As shown in Figure 3, twenty histological images in 24-bit RGB colour were obtained by digitizing several sections from this specimen at different regions with an objective magnification of 20x. The size of each image is 1280 × 800.

k-Means++ [23], GMM-EM, Mean Shift, Kernel Graph-cuts (KGC) [18], hidden Markov Random Field with Expectation-Minimization algorithm (HMRF-EM) [19] and the proposed FMShift method had been implemented using MATLAB (The MathWorks, Inc., Natick, MA, USA), and were employed for histological image segmentation. a* and b* colour components of histological images were used as input features for previous three methods and FMShift while RGB for KGC and HMRF-EM. For the convenience of comparisons, the number of clusters *k* was given in prior. The clustering results of FMShift and Mean Shift were sorted by the number of assigned points in descent order. Then only the top *k* clusters were kept and others were reassigned into the nearest cluster. Considering that the objective of the segmentation is to identify fibrosis and vessels from other liver tissues, we set *k =* 3 in all experiments. In FMShift method, we set the parameter *e* to one thousandth of maximum range of a* and b* colour components, and *h* to 40 times *e*. Radial basis function with σ = 0.5 was used as the kernel function in KGC method. And the number of iterations of EM algorithm in HMRF-EM and GMM-EM is limited up to 100.

For quantitative comparison, we took manually segmentation results as references, and employed three performance metrics: Dice index, Rand index [24] and Variation of Information [25]. Dice and Rand indexes were used to measure the similarity between segmentations obtained by using different methods and their corresponding references. Denote *S* the segmentation result and *R* the reference. For a given pixel *x*_*i*_ of the segmented image, it is labelled with *l*_*i*_ and *l’*_*i*_ respectively in *S* and *R*. The Dice Index (DI) is defined as the size of the intersection divided by the average size of *S* and *R* as follows

11 $$ DI\left(S,R\right)=\frac{2\left|S\cap R\right|}{\left|S\right|+\left|R\right|}. $$

And the Rand Index (RI) is defined as the ratio of the number of pairs of pixels which have a compatible label relationship between *S* and *R*, and is computed as follows:

12 $$ RI\left(S,R\right)=\frac{1}{\left(\begin{array}{c}\hfill N\hfill \\ {}\hfill 2\hfill \end{array}\right)}{\displaystyle \sum_{\begin{array}{c}\hfill i,j\hfill \\ {}\hfill i\ne j\hfill \end{array}}\left[\mathbf{I} \left({l}_i={l}_j\wedge {l}_i^{\prime }={l}_j^{\prime}\right)+\mathbf{I} \left({l}_i\ne {l}_j\wedge {l}_i^{\prime}\ne {l}_j^{\prime}\right)\right]}. $$

where **I** is the identity function. The Variation of Information (VoI) [25] is an information-based performance metric and can be used to measure the distance between *S* and *R*. VoI satisfies

13 $$ VoI\left(R,S\right)=H(R)+H(S)-2I\left(R,S\right), $$

where *H*(*X*) is the entropy of *X*, and *I*(*X*,*Y*) is the mutual information between *X* and *Y*. Larger values of DI and RI and a smaller value of VoI mean higher segmentation accuracy.

**Figure 4:**
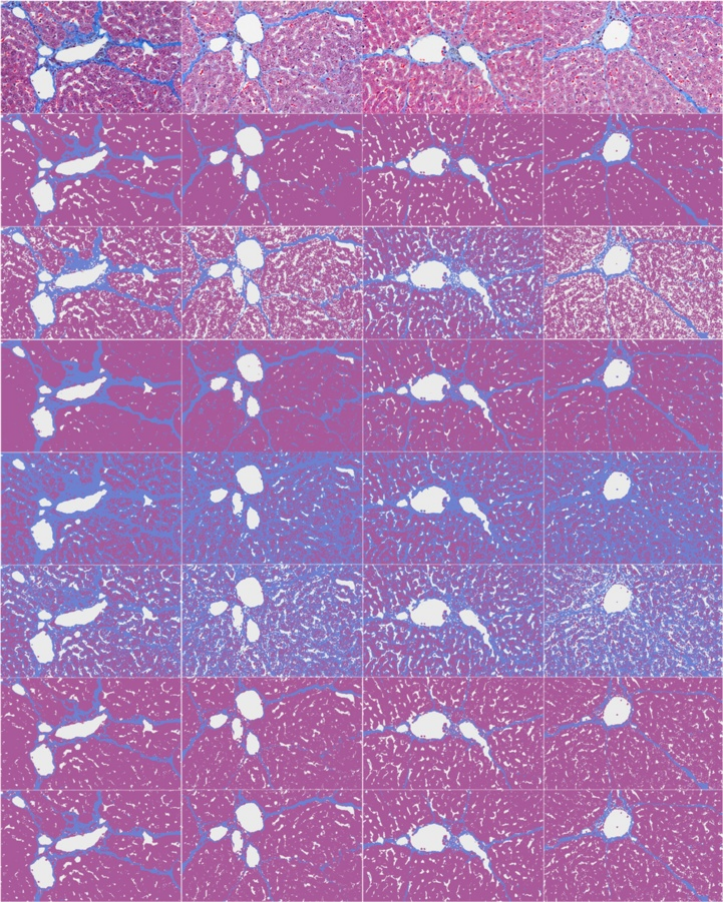
**Segmentation results of four liver fibrosis histological imageS by using k-Means, GMM-EM, HMRF-EM, KGC, Mean Shift, and FMShift.** Four columns are corresponding to four histological images. Fibrosis, vessels and other tissues are represented in light blue, white and lavender respectively. From top to bottom are original histological images, manual segmentations, and segmentations using k-Means, GMM-EM, HMRF-EM, KGC, Mean Shift and the proposed FMShist method.

Figure 4 shows the segmentation results of four histological images selected from the dataset by using k-Means++, GMM-EM, KGC, HMRF-EM, Mean Shift and our proposed FMShift respectively. Fibrosis, vessels (including sinusoids) and other tissues are represented in light blue, white and lavender respectively. Table 1 illustrates the average accuracies with standard deviation over segmentation results obtained from twenty histological images using different methods. The performance of six segmentation methods is quantified by Rand Index and Variation of Information for global evaluations. And the segmentation results of fibrosis and vessels are also measured by Dice Index independently at the same time. Table 2 summaries the average computation times for each method. All the above results are obtained with a standard Windows computer equipped with a 2.4 GHz Intel Core i5 processor and 8 GB RAM.

**Table 1:** **Comparison of segmentation accuracies**

**Method**	**Dice index**		**Rand index**	**Variation of information**
	**Fibrosis**	**Vessels**		
k-Means	0.67 ± 0.24	0.52 ± 0.24	0.69 ± 0.08	1.25 ± 0.20
GMM-EM	0.60 ± 0.13	0.68 ± 0.22	0.76 ± 0.10	1.03 ± 0.29
HMRF-EM	0.28 ± 0.14	0.76 ± 0.22	0.62 ± 0.10	1.35 ± 0.24
KGC	0.23 ± 0.10	0.69 ± 0.19	0.55 ± 0.08	1.62 ± 0.16
Mean Shift	0.87 ± 0.05	0.84 ± 0.09	0.91 ± 0.03	0.53 ± 0.12
FMShift	0.86 ± 0.05	0.84 ± 0.07	0.91 ± 0.02	0.54 ± 0.12

**Table 2:** **Comparison of average computation times**

**Method**	**k-Means++**	**GMM-EM**	**HMRF-EM**	**KGC**	**Mean Shift**	**FMShift**
**Avg. times (sec.)**	4.2	117.0	954.2	14.8	732.3	6.6

It is shown in Figure 4 that the tissues adjacent to collagen tend to be wrong segmented as fibrosis using HMRF-EM and KGC. The possible reason is that the shape of collagen is thin and elongated. Graph cut based image segmentation method has the problems to segment them due to shrinkage bias, and HMRF-EM uses spatial information through the mutual influences of neighbours. So it results in small values of Dice Indexes for fibrosis segmentation by using HMRF-EM and KGC methods and also decreases the Rand Indexes as listed in Table 1. At the same time, k-Means method can successfully detect spherical clusters with similar size but is not a good scheme for others. This limitation makes it easy to assign points to wrong clusters. Thus the tissues adjacent to sinusoids are segmented wrong by k-Means++ as shown in Figure 4. GMM-EM is based Gaussian mixture models and is sensitive to the distribution of feature points. However, this requirement is usually hard to meet in the case of histological image segmentation. So GMM-EM does not achieve high Dice and Rand indexes as shown in Table 1.

The quantitative results listed in Tables 1 and 2 show that the speed of FMShift method is comparable to that of k-Means++ and much faster than other schemes including standard Mean Shift clustering approach while the segmentation accuracies obtained by FMShift and Mean Shift are almost the same but much better than that of other methods. By estimating the probability density distribution in advance and employing the integral image scheme, FMShift reduces the computation cost of standard Mean Shift clustering significantly. And because histological images are stained manually, the range of colour components is restricted. This makes the frequency matrix *w* sparse and also speeds it up to find the density peaks in FMShift. The above reasons make FMShift a very fast approach even in handling large-scale histological image segmentation problems. At the same time, FMShift is Mean Shift scheme based. So it has no demands on the shape of underlying distribution and thus achieves high accuracies in liver histological image segmentation.

## Conclusions

We have developed a histological image segmentation approach by employing improved Mean Shift clustering. To eliminate illumination variations, colour histological image is transformed into CIE L*a*b* colour space, and then a* and b* components are extracted as features for clustering. The clustering approach consists of three steps. In the first step, the probability density distribution is estimated by splitting the effective feature space located with observations into tiny squares and computing the frequencies of observations occurring in each square. The second step is to separate the feature space into different regions by employing Mean Shift scheme to finding density peaks. Then all observations are assigned into different clusters according to which square they fall into in the last step. And an integral image scheme is used to speed up the computation of mean shift vector at the same time. By employing the probability density estimation and integral scheme, the computation cost of standard Mean Shift clustering method is significantly reduced while keeping the accuracy the same. From the results of numerical experiments on liver fibrosis histological images, we have the conclusion that the proposed method is a fast and reliable approach for color image segmentation, especially for large-scale histological image segmentation.

In this paper, we only use two dimensional color features of histological images and discuss how to accelerate Mean Shift method in the two-dimension case. Our future work includes extending fast Mean Shift scheme to high dimensional case and taking more features, such as shape and texture, into consideration for better performance.
